# A Study of the Miliary Plaques Found in Brains of the Aged

**Published:** 1912-08

**Authors:** Solomon C. Fuller

**Affiliations:** From the Pathological Laboratory, Westborough State Hospital, Mass.


### A Study of the Miliary Plaques Found in Brains of the Aged

By SOLOMON C. FULLER, M.D.
(From the Pathological Laboratory, Westborough State Hospital, Mass.)

From the *American Journal of Insanity*, October, 1911

Miliary plaques, similar to the findings reported by Redlich in 1898, and by Blocq and Marinesco in 1892, were found by Fuller in the brains of 16 cases out of 33 elderly subjects dying insane, and in 1 case out of 6 brains from elderly subjects without psychoses. As a control he examined the material from 50 younger subjects dying of various mental diseases, but without finding the plaques.

The general morphology of the plaques is best studied by the Bielchowskys silver impregnation method. The plaques are described as discreet structures of variable size in which a dark, circular, homogeneous, nuclear-like mass is centrally disposed. Surrounding the dark, homogeneous portion is an area of variable extent, always larger and lighter than the nuclear portion and darger than the adjacent brain tissue. The diameter of the plaques may be 4 to 6 times that of a ganglion cell.

When the plaques are present they are usually widely distributed throughout the cerebrum, but with a preponderance in the frontal lobes and hippocampal regions.

Fuller believes the plaques are the deposited products of pathological metabolism resulting from degenerating nervous elements (fibrils). The views which other writers hold regarding these plaques are also discussed. Fischer considers them characteristic of presbyophsenia while Huebner denies they are characteristic of any special psychosis, merely indicating that the patient has reached the 5th decade. Fuller concludes that they cannot be considered as characteristic for any special form of mental disease, autho occurring with greater frequency in senile dementia than in any other form of insanity.

The article is illustrated with 17 photomicrographic plates, and there is an extensive list of references attached.

